# Tailoring the Luminescence Properties of Silver Clusters Confined in Faujasite Zeolite through Framework Modification

**DOI:** 10.3390/ma15217431

**Published:** 2022-10-23

**Authors:** Xinling Xv, Song Ye, Ling Pan, Peixuan Lin, Huazhen Liao, Deping Wang

**Affiliations:** School of Materials Science and Engineering, Tongji University, Shanghai 201804, China

**Keywords:** silver nanocluster, luminescence properties, desilication and dealumination, faujasite zeolite, XPS study

## Abstract

Faujasite zeolites with a regular micropore and mesopore structure have been considered desirable scaffolds to stabilize luminescent silver nanoclusters (Ag CLs), while turning of the emission properties of the confined Ag CLs is still under investigation. In this study, the desilicated and dealuminated faujasite zeolites were first prepared to modify the zeolite framework and Si/Al ratio before Ag^+^ loading. With thermal treatment on the thereafter Ag^+^-exchanged zeolites, the Ag CLs formatted inside the D6r cages showed red-shifted emission in the desilicated zeolites and blue-shifted emission in the dealuminated zeolites, so that a tunable emission in the wavelength range of 482–528 nm could be obtained. Meanwhile, the full width at half maximum of the emission spectra is also closely related with framework modification, which monotonously increases with enhancing Si/Al ratio of host zeolite. The XRD, XPS, and spectral measurements indicated that the tunable luminescence properties of Ag CLs result from the controlling of local crystal field and coupling between host lattice and luminescent center. This paper proposes an effective strategy to manipulate the emission properties of Ag CLs confined inside zeolites and may benefit the applications of noble metal clusters activated phosphors in imaging and tunable emission.

## 1. Introduction

Luminescent Ag clusters (Ag CLs) that consist of several silver ions and silver atoms have attracted wide research interest for photocatalysis, imaging, detection, and illumination applications due to their remarkable optical properties including strong absorption of UV light, tunable emission in the visible region, large Stokes shifts, and high quantum efficiency approaching commercial phosphors [1,2,3]. However, Ag CLs easily aggregate into larger silver nanoparticles (Ag NPs) due to their high surface energy and thus lose radiative emission ability [4,5]. In recent studies, organic compounds such as DNA, colloids, and sols; and inorganic compounds, such as zeolites and glasses, have been applied to stabilize Ag CLs [6,7,8]. Compared with amorphous glasses with a rigid structure, the microporous aluminosilicate zeolites, which are composed of framework TO_4_ tetrahedra (T = Si, Al) linked to each other by oxygen atoms, show advantages as scaffolds for Ag CLs. The defined cages and efficient cation exchange capacity of zeolites are beneficial to readily controlling Ag CLs’ size and chemical state [9,10].

The emissive properties of the Ag CLs confined inside zeolites are greatly dependent on the framework topology, internal porous size, and extraframework cation type of the host zeolites as well as Ag^+^-loading degree and subsequent thermal treatment condition. So far, various strategies have been adopted to adjust the local environment of Ag CLs for achieving tunable emission [11,12,13,14]. For example, the emission of Ag CLs confined in faujasite zeolites turned from blue to green and yellow with a decreasing Si/Al ratio [15,16]. Due to the change in the local crystal field resulting from different radii and electronic configurations of balancing cations, the tunable emission of Ag CLs can be realized through incorporation with different extraframework cations such as Li^+^, Na^+^, K^+^, and Ca^2+^ before Ag^+^ loading [17,18,19]. Previous studies suggested that the emission peak wavelength of Ag CLs shows red shift with increasing Ag^+^-exchanged amount and thus is considered as an alternative method to manipulate the emission properties of Ag CLs [20]. Moreover, proper thermal treatment on the Ag^+^-exchanged zeolites benefited the generation of strong emission due to the efficient formation of Ag CLs and the avoidance of zeolite structure deconstruction [21,22]. The above studies indicated zeolites are desirable hosts for Ag CLs to manipulate their optical properties for diverse applications.

So far, the synthesis of Ag CLs-loaded zeolites is mainly based on the extraframework cation exchange in AgNO_3_ aqueous solution. Because the SiO_4_ tetrahedra is electrically neutral while AlO_4_ tetrahedra is negatively charged and balanced by extraframework cations such as Li^+^, Na^+^, and NH_4_^+^, the exchangeable site for Ag^+^ largely depends on the framework Si/Al ratio [23,24]. The zeolites with low Si/Al ratio are suitable for Ag^+^-loading through ion-exchanged methods, such as Linda A and faujasite types, which have enough exchangeable sites for the intake of Ag^+^ and thus benefit the formation of luminescent Ag CLs to achieve remarkable emission properties [25,26,27]. Conversely, the zeolites with high Si/Al ratio have few cation-exchange sites and are not desirable for the growth of Ag CLs but may benefit the loading of quantum dots or nanocrystals [28,29]. As the luminescence properties of Ag CLs strongly depend on the local crystal field of host zeolite and Ag^+^-loading amount-related chemical states, it is necessary to understand the interaction between the luminescence center and host zeolite. Previous studies mainly focused on the influence of extraframework cations on the luminescence properties of Ag CLs, while the modification of Si/Al ratio provides a new idea for the regulation and optimization of Ag CLs luminescence performance in zeolites [17,18].

In this study, by taking faujasite Y as the parent zeolite, the framework desilication and dealumination were first conducted with sodium hydroxide, hydrochloric acid, and oxalic acid treatments before Ag^+^ loading. After thermal treatment on the Ag^+^-exchanged zeolites, the formatted Ag CLs showed tunable emission in the wavelength range of 482–528 nm. Meanwhile, the full width at half maximum Ag CLs emission spectra monotonously increased with the increasing Si/Al ratio of host zeolite. The tunable luminescence property of Ag CLs confined inside zeolite was studied based on the XRD, XPS, and spectral measurements.

## 2. Materials and Methods

### 2.1. Reagents

Sodium form of zeolite Y ((Na_6.5_^+^) [Al_6.5_Si_17.5_O_48_]) with SiO_2_/Al_2_O_3_ = 5.1 was obtained from Alfa Aesar, Shanghai, China. Flake sodium hydroxide (NaOH, AR), hydrochloric acid (HCl, 37%), oxalic acid (OA, 98%, anhydrous), and silver nitrate (AgNO_3_, 99.8%) were purchased from Sinopharm Chemical Reagent Co., Ltd., Shanghai, China. In this study, all reagents and materials were used without further purification.

### 2.2. Desilication on FAU Zeolite

Each 1 g of the parent zeolite (NaY) was stirred in 30 mL of NaOH aqueous solution with different concentrations of 1.0 and 2.0 mol/L for 70 min at 75 °C. Subsequently, the resulting suspensions were cooled to room temperature, filtered, washed with deionized water three times, and then the solid products were dried in an oven at 60 °C for 24 h. The alkali-treated zeolites were denoted as DSiY1 and DSiY2 according to the NaOH aqueous solution concentrations.

### 2.3. Dealumination on FAU Zeolite

Each 1 g of the NaY zeolite was stirred in 30 mL of OA solution with different concentrations of 0.01, 0.03, and 0.05 mol/L at room temperature for 1 h. Meanwhile, each 1 g of the NaY was stirred in 30 mL of 0.1 mol/L HCl solution at 70 °C for 3 h. Afterwards, the resulting suspensions were filtered and washed with deionized water three times, and then the solid products were dried in an oven at 60 °C for 24 h. The samples were designated as DAlYO1, DAlYO3, and DAlYO5 for the OA treated zeolites, separately, according to the OA solution concentrations, and DAlYH for the HCl treated zeolites.

### 2.4. Preparing of Ag^+^-Exchanged Zeolites

Each 0.5 g of the parent zeolite, and desilicated and dealuminated zeolites was immersed into 50 mL of AgNO_3_ aqueous solution with the concentration of 10 mmol/L. The mixtures were stirred for 1.5 h under dark conditions at 40 °C. Then, the precipitated materials were washed with deionized water three times and dried in an oven at 60 °C for 24 h before being calcined at 600 °C for 2 h. The obtained silver-loaded zeolites were named NaY-Ag, DSiY1-Ag, DSiY2-Ag, DAlYO1-Ag, DAlYO3-Ag, DAlYO5-Ag, and DAlYH-Ag. Moreover, the same amount of AgNO_3_ aqueous solution with concentrations of 5, 20, and 30 mmol/L was also used as exchanged solution for the above zeolites to compare the luminescence performance of Ag CLs with different Ag^+^-loading amounts.

### 2.5. Measurements

X-ray powder diffraction (XRD) analyses were conducted using a Rigaku Smartlab9 diffractometer with monochromated Cu-Kα radiation (λ = 1.5406 Å, 40 kV, 20 mA) (Tokyo, Japan). The transmission electron microscopy (TEM) measurement was carried out using a JEOL2100F (Tokyo, Japan) with acceleration voltage of 200 kV. X-ray photoelectron spectroscopy (XPS) measurements were conducted on a Thermo ESCALAB 250XI system (Waltham, MA, USA,) and the Al Kα radiation (hυ = 1486.6 eV) with a beam spot of 650 μm was used as an X-ray source. The pass energies for survey spectra and high-resolution spectra were 100 eV and 30 eV, respectively. The photoluminescence (PL) and the photoluminescence of excitation (PLE) spectra were performed on a fluorescence spectrometer (Edinburgh FLS980, Edinburgh, UK) with a 450 W xenon lamp as the steady-state excitation source, double grating monochromators for both excitation and emission compartments, and a Hamamatsu R928P thermoelectrically cooled photomultiplier tube for detection, operating in right-angle geometry mode with automatic spectrum correction function. Each synthesized powder sample was loaded into a circular holder, and fluorescence was measured with 240–360 nm excitation filter and 400–650 nm emission filter with 2.4 nm spectral slit widths and 2 nm step at room temperature. Measuring parameters such as width of monochromatic slit, photomultiplier tube (PMT) detector voltage, scan speed, and spectral resolution were kept constant throughout the analysis of materials. The diffuse reflectance spectra measurements were carried out on a Shimadzu UV-3600i Plus spectrophotometer (Kyoto, Japan) with an integrating sphere attachment, in which all diffuse light was collected and focused to reach the detector, in the scanning wavelength range of 200–600 nm with a step of 1 nm and a scan rate of 600 nm/min by contrasting the reference (BaSO_4_). The reflectance spectra were plotted in pseudo-absorbance mode using the Kubelka–Munk transform.

## 3. Results and Discussion

**Figure 1:**
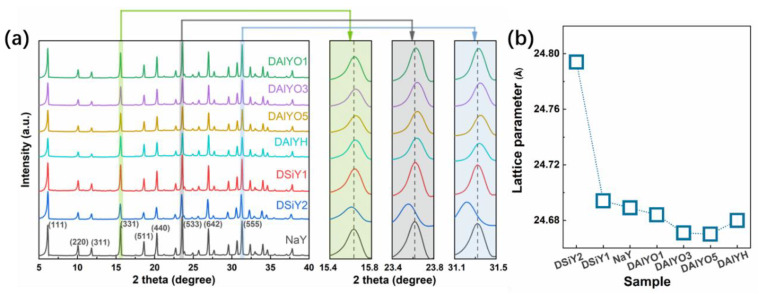
XRD patterns of NaY, DSiY1, DSiY2, DAlYO1, DAlYO3, DAlYO5, and DAlYH, (**a**), and the corresponding calculated unit cell parameters (**b**). The diffraction peaks of the parent NaY zeolite were indexed according to standard PDF#43-0168.

The structure of NaOH desilicated, and OA and HCl dealuminated zeolites were first checked with XRD measurement together with the parent NaY zeolite. As shown in Figure 1a, the desilicated and dealuminated zeolites exhibit the same diffraction peaks with the parent NaY zeolite but in lower diffraction intensities, indicating the applied chemical leaching did not cause obvious structure destruction but resulted in reduced relative crystallinity. Compared with NaY, the diffraction peaks of DSiY1 and DSiY2 show small-angle shifts, while the diffraction peaks of DAlYO1, DAlYO3, DAlYO5, and DAlYH exhibit large-angle shifts. The lattice parameters were calculated according to 2dsinθ=nλ, where *λ* is the wavelength of X-ray, *n* is the order of diffraction, *d* is the distance between layers of atoms, and *θ* is the angle of incidence of the incoming X-ray. As shown in Figure 1b, the lattice parameters exhibit monotonously increased or decreased values with increasing NaOH or OA concentration. The unit cell expansion and contraction suggest the selective extraction of framework Si or Al, respectively, owing to the fact that the T-O-T bond for aluminate (~1.75 Å) is longer than that for silicon (~1.62 Å) [30]. The lattice parameter (LP) variation in LP_DSiY2_ > LP_DSiY1_ > LP_NaY_ > LP_DAlYO1_ > LP_DAlYO3_ > LP_DAlYO5_ shown in Figure 1b is in agreement with the deeper removal of Si or Al from the framework with increasing NaOH or OA concentration, respectively.

**Figure 2:**
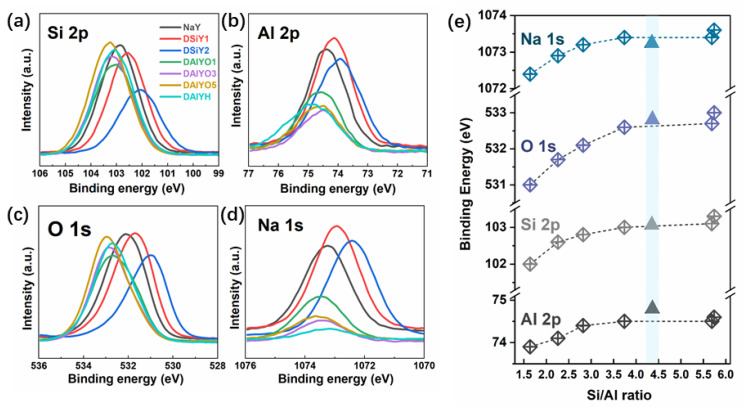
XPS spectra for Si 2p (**a**), Al 2p (**b**), O 1s (**c**), and Na 1s (**d**) in NaY, DSiY1, DSiY2, DAlYO1, DAlYO3, DAlYO5, and DAlYH. Summary of the Si/Al ratio dependence of BE, in which the triangles represent for the data for DAlYH (**e**).

The XPS measurements were carried out to study the influence of acid and alkali leaching on the elemental composition and chemical state of the parent NaY zeolite. Figure 2a–d show the high-resolution XPS spectra of Si 2p, Al 2p, O 1s, and Na 1s, respectively, based on which the atomic percentages were first calculated and are shown in Table 1. The decreased Si/Al ratio in desilicated zeolites and the increased Si/Al ratio in dealuminated zeolites also suggested the successful removal of Si and Al. The extraction of Si or Al should both lead to increased relative atomic percentages for all the other chemical elements. However, the Na content exhibited an inverse relationship with Si/Al ratio after the leaching of Al with OA treatment, which decreased with increasing dealumination degree; this could be attributed to the simultaneous removal of extra-framework cations of Na^+^ together with the framework Al. However, as shown in Table 1, the Si/Al ratio and Na content of DAlYH were both lower than those of DAlYO3 and DAlYO5. This is due to the fact that OA treatment extracted both the tetrahedral framework Al and the octahedral nonframework Al, while HCl treatment mostly extracts the framework Al, and the removal of non-framework Al will not lead to reduced Na [31,32,33]. The dealumination process with OA treatment involves two steps: the hydrolysis of aluminum from the framework owing to the high acidity of the solution, followed by the chelation of each aluminum ion with three oxalic acid molecules [34,35]. Due to the mild sodium hydroxide and hydrochloric/oxalic acid treatments, the morphologies of the resulting zeolites were well-kept, and no obvious corrosion could be observed from the TEM images (see Figure S1 in ESI).

Furthermore, the high-resolution XPS spectra in Figure 2 indicate that the photoelectron peaks of Si 2p, Al 2p, O 1s, and Na 1s all showed negative shifts in DSiY1 and DSiY2, while positive shifts in DAlYO1, DAlYO3, DAlYO5, and DAlYH compared with in the parent NaY zeolite. This is in accordance with previous reports that the binding energy (BE) of each zeolite element shifts in the same direction with the change in the Si/Al ratio [36,37]. Figure 2e summarizes the binding energies of Si 2p, Al 2p, O 1s, and Na 1s in all the considered zeolites. The chemical shift strongly depends on the Si/Al ratio and increases with increasing Si/Al ratio in the order of O 1s > Si 2p > Na 1s > Al 2p. Generally, the BE shifts can be explained from the charge transfers in zeolite lattice point-of-view; the easier it is to remove an electron under X-ray irradiation, the lower the binding energy [14,38]. The framework desilication results in the removal of Si atoms and the breakage of Si-O-Si bonds, accompanied by the increased proportion of Si-O-Al bonds. Therefore, the negative BE shifts in Si 2p, Al 2p, O 1s, and Na 1s for those desilicated zeolites with decreasing Si/Al ratio could be attributed to the increased negative charge on the framework caused by the increased Si-O-Al species [39,40]. Similarly, dealumination led to the decreased Si-O-Al proportion and, therefore, resulted in positive BE shifts in Si 2p, Al 2p, O 1s, and Na 1s with increasing Si/Al ratio. Particularly, DAlYH exhibited more remarkable positively shifted BE of Al compared with that of the OA dealuminated zeolites, which could be attributed to the increased Al-O-H and Si-O-H proportions. On dealumination with the strong acid of HCl, a larger amount of Na^+^ involved in O-Na ionic bonding environments was replaced by H^+^ to form Al-O-H and Si-O-H groups [38,41]. As H^+^ has a higher electronegativity than Na^+^, the aluminate in Al-O-H and the nonbridging oxygen in Si-O-H showed stronger BE. Moreover, the more significant BE change observed in Si 2p (ΔE = 1.3 eV) compared with that of Al 2p (ΔE = 0.7 eV) could be explained by the fact that the oxygen in an oxide prefers the ionic status to the covalent one; therefore, the more ionic Al induced a large shift in Si 2p binding energy in the Si-O-Al structure for zeolites with low Si/Al ratio [40].

**Figure 3:**
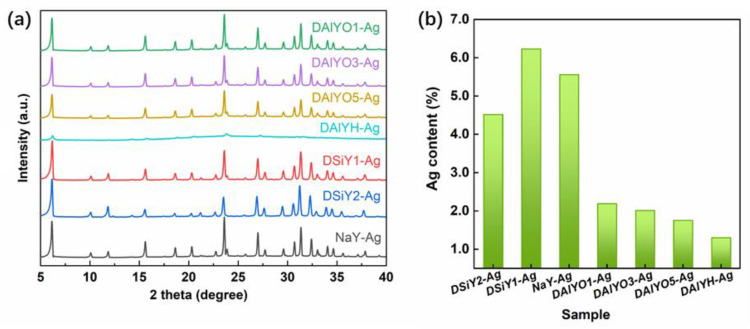
XRD patterns of NaY-Ag, DSiY1-Ag, DSiY2-Ag, DAlYO1-Ag, DAlYO3-Ag, DAlYO5-Ag and DAlYH-Ag (**a**); Ag atomic content from XPS measurement (**b**).

The XRD patterns for the Ag^+^-exchanged zeolites are shown in Figure 3a. It can be seen that the Ag^+^ exchange and the afterward thermal treatment did not lead to structure destruction, except for DAlYH-Ag. In contrast, the XRD pattern for DAlYH-Ag shows extremely weak diffraction peaks. This approaching amorphous structure of DAlYH-Ag was due to the extraction of a high degree of framework Al together with Na^+^, which reduced the thermal stability of host zeolite [42,43]. Figure 3b shows the atomic percentage of Ag, which drastically decreased from 5.56% in NaY-Ag to 2.19%, 2.01%, 1.75%, and 1.3% in DAlYO1-Ag, DAlYO3-Ag, DAlYO5-Ag, and DAlYH-Ag, respectively. This was reasonable because dealumination simultaneously reduced exchangeable Na^+^ sites; therefore, less Ag^+^ could be exchanged into the zeolite lattice with increasing dealumination degree. Differently, the Ag atomic percentage in DSiY1-Ag was slightly higher than that in NaY, which resulted from the increased relative Na content (exchangeable Na^+^ sites) in the desilicated zeolites (Table S1). The lower Ag atomic content in DSiY2-Ag was due to the reduced Ag^+^-Na^+^ exchange efficiency caused by desilication. The above XPS results suggest that the framework modification through desilicated or dealuminated processes influences the exchange rate of Ag^+^.

**Figure 4:**
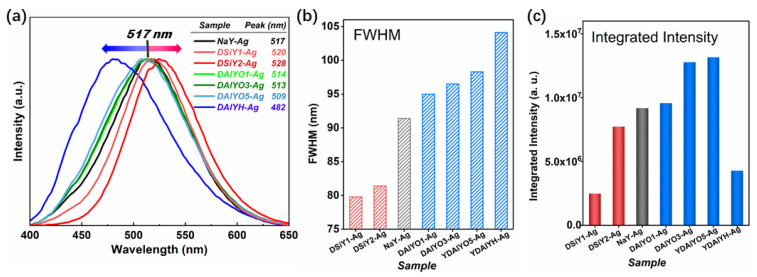
Normalized PL spectra (**a**), FWHM of the PL spectra (**b**), and integrated intensities (**c**) for NaY-Ag, DSiY1-Ag, DSiY2-Ag, DAlYO1-Ag, DAlYO3-Ag, DAlYO5-Ag, and DAlYH-Ag.

Regarding the Ag^+^-Na^+^ exchange and afterward heat treatment, Ag CLs were formed inside the double six ring (D6r) of FAUY zeolites, which gave off bright visible emission under UV excitation (see Figure S2 in ESI) [16,22]. Figure 4a shows the normalized PL spectra of Ag CLs with the most efficient 311 nm excitation (see Figure S3 in ESI). The emission peak of Ag CLs located at 517 nm in NaY, which red-shifted to 520 and 528 nm in DSiY1-Ag and DSiY2-Ag, respectively, while blue-shifted to 514, 513, 509, and 482 nm in DAlYO1-Ag, DAlYO3-Ag, DAlYO5-Ag, and DAlYH-Ag, respectively. The spectra results indicated a tunable emission range of Ag CLs from 482 to 528 nm through framework modification. Furthermore, the full width at half maximum (FWHM) of Ag CLs also showed Si/Al ratio dependency, which monotonously decreased with ascending desilicated degree or increased with ascending dealuminated degree. The red-shifted emission of Ag CLs and the reduced FWHM of the emission spectra with increasing desilicated degree indicate reduced energy splitting between the excited state and ground state of Ag CLs caused by decreased local crystal field and weaker coupling between the host lattice and luminescent center, which could be attributed to the expanded unit cell in the desilicated zeolites [19,44]. Similarly, the blue-shifted emission of Ag CLs and the increased FWHM of the emission spectra with increasing dealumination degree were due to increased local crystal field and stronger coupling between host lattice and luminescent center because of unit cell contraction. In addition, compared with DAlYO1-Ag, DAlYO3-Ag, and DAlYO5-Ag, the drastically blue-shifted emission and increased FWHM of DAlYH-Ag could be attributed to the amorphous state of the host (Figure 3a).

**Figure 5:**
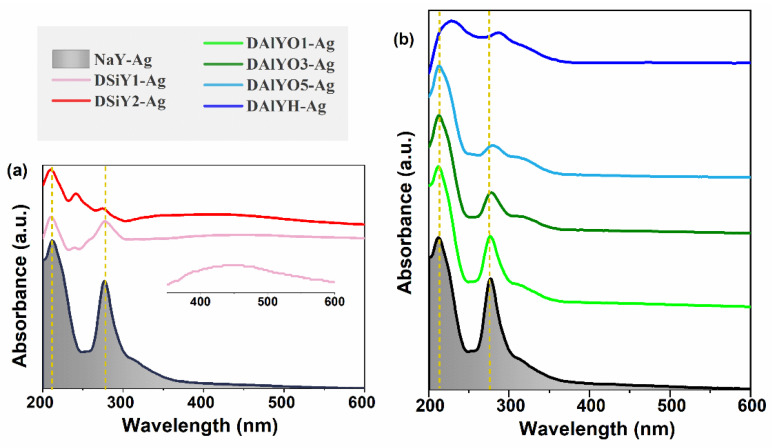
Reflectance spectra plotted in pseudo-absorbance mode for NaY-Ag, DSiY1-Ag, DSiY2-Ag (**a**), NaY-Ag, DAlYO1-Ag, DAlYO3-Ag, DAlYO5-Ag, and DAlYH-Ag (**b**).

Moreover, framework modification through desilication or dealumination also manipulated the emission intensity of Ag CLs, as indicated by the integrated emission intensities in Figure 4c. In order to study the influence of Ag chemical states on the luminescence properties of Ag CLs, the absorption spectra were measured and compared. As shown in Figure 5a, the absorption band of NaY-Ag at 210 nm could be assigned to the 4d^10^→4d^9^5s^1^ electronic transition of the separated Ag^+^ in the zeolite framework, and the absorption band at approximately 270 nm originated from the postively charged Ag CLs, which was confined inside the FAUY zeolite [14,19]. According to the works from Chebbi and Azambre, the silver species that bear a positive charge have a shorter wavelength absorption than the neutral Ag_n_^0^ clusters [45,46]. The absorption intensity of Ag^+^ and Ag CLs both decreased with increasing desilication degree, and the surface plasma resonance (SPR) absorption band located around 400 nm could be observed in DSiY1-Ag and DSiY2-Ag, indicating the formation of bigger Ag NPs. This was reasonable because desilication leads to the formation of mesopores, which benefit the aggregation of Ag CLs [47,48]. Therefore, the monotonously decreased Ag CLs emission intensity after desilication resulted from the formation of nonluminescent Ag NPs. On the other hand, both Ag^+^ absorption and Ag CLs absorption decreased with increasing dealumination degree, while no SPR peak could be observed, as shown in Figure 5b, which is in accordance with the XPS results that less Ag^+^ ions were exchanged into host zeolite due to the reduced exchangeable sites. Interestingly, the emission intensity of Ag CLs monotonously increased with dealumination degree with OA treatment due to the suppression of concentration quenching. Moreover, the absorption peaks for DAlYH-Ag broadened and red-shifted to 228 and 288 nm because of the collapse of the zeolite framework.

**Figure 6:**
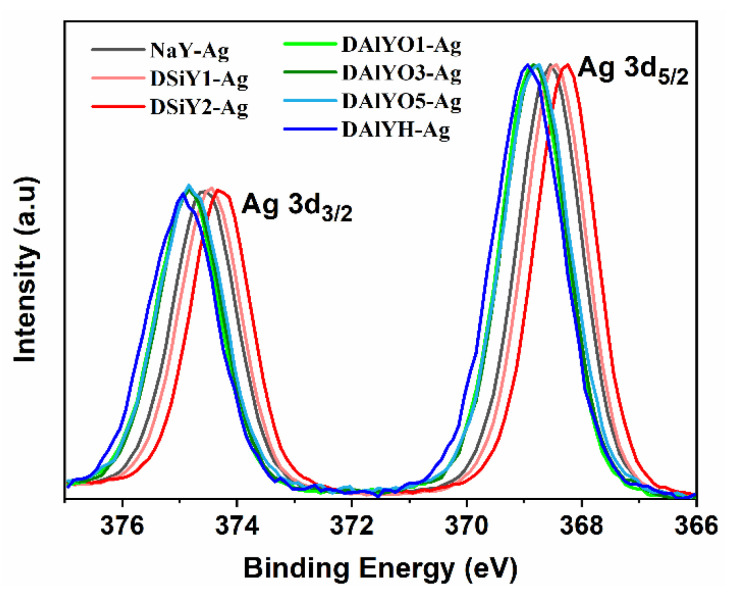
Normalized XPS spectra of Ag 3d in NaY-Ag, DSiY1-Ag, DSiY2-Ag, DAlYO1-Ag, DAlYO3-Ag, DAlYO5-Ag, and DAlYH-Ag.

XPS measurements were also carried out to study the chemical state and chemical shift of Ag CLs, as can be observed from Figure 6; the high-resolution XPS spectra of Ag 3d showed two photoelectron peaks corresponding to Ag 3d_3/2_ and Ag 3d_5/2_. The binding energies (BE) of Ag 3d_3/2_ and Ag 3d_5/2_ in Ag^+^-exchanged zeolites showed positive shift with increased framework aluminum extraction degree, but negative shift with desilication degree. The monotonously increased BE up to 0.7 eV (Table S2) with increasing Si/Al ratio indicated the Ag CLs became less ionic, and it was harder to extract the electron from Ag. Previous studies also indicated that ionic silver has a higher BE of Ag 3d_5/2_ than metallic Ag^0^ [5,14,49]; therefore, the decreased BE in DSiY1-Ag and DSiY2-Ag compared with that in NaY-Ag suggested the formation of metallic Ag NPs, which was also in accordance with the absorption spectra.

**Figure 7:**
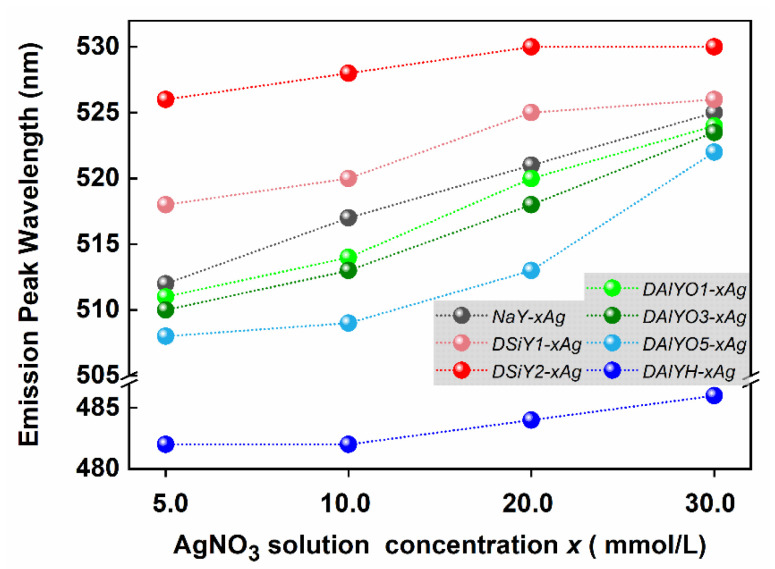
Summary of Ag CLs emission peak wavelength in FAUY and desilicated and dealuminated FAUY zeolite with increasing Ag^+^-exchanged amount.

The emission wavelength of Ag CLs confined inside zeolites is closely related with Ag^+^-exchanged amount, and a red-shifted emission can generally be obtained if the AgNO_3_ exchange solution concentration is increased while fixing other experimental parameters [5,15,20]. Herein, we prepared a series of control samples with different Ag^+^-exchanged amounts by using the parent NaY zeolite; desilicated DSiY1 and DSiY2 zeolites; and dealuminated DAlYO1, DAlYO3, DAlYO5, and DAlYH zeolites as hosts. The emission peak wavelengths of Ag CLs in those zeolites are summarized in Figure 7, which also showed red-shift with increasing Ag^+^-exchanged amount as reported before. By solely adjusting AgNO_3_ exchange solution concentrations, the emission peak wavelength of Ag CLs in NaY zeolite could only be turned in the range of 512–525 nm, while framework modification allowed an extended tunable range of 482–528 nm.

## 4. Conclusions

In this study, NaY zeolite was first treated with sodium hydroxide, hydrochloric acid, and oxalic acid solutions to selectively leach Si and Al. After Ag^+^-Na^+^ exchange and thermal treatment, the luminescent Ag CLs were formatted inside the D6r of the host zeolite, which gave off bright emission under UV excitation. The spectral studies indicated that through framework modification, the emission peak wavelength of Ag CLs could be turned from 482 to 528 nm. This is because desilication and dealumination could effectively modify the lattice parameter of FAUY zeolite host and, therefore, exert varied local crystal field of Ag CLs and coupling between host lattice and luminescent center. Further XPS studies indicated that desilication and dealumination influenced the exchangeable site amount, the exchanged rate of Ag^+^, and the chemical state of Ag CLs. Compared with solely adjusting the Ag^+^ exchange amount, the framework modification could tailor the Ag CLs emissions in a wider wavelength region with improved emission intensity. This study proposed an effective strategy to manipulate the emission properties of Ag CLs confined in zeolites and may benefit the applications of noble metal clusters activated phosphors in imaging and tunable emission.

## Figures and Tables

**Table 1:** XPS data of elemental composition of the parent NaY zeolite, and the desilicated and dealuminated zeolites.

Sample	XPS Atomic (%)				Si/Al
	Si	Al	O	Na	
NaY	21.15	7.50	61.85	9.50	2.82
DSiY1	19.33	8.57	60.86	11.24	2.26
DSiY2	16.70	10.16	59.61	13.53	1.64
DAlYO1	23.13	6.18	64.89	5.80	3.74
DAlYO3	25.33	4.45	67.33	2.89	5.69
DAlYO5	25.65	4.47	66.97	2.91	5.74
DAlYH	24.92	5.66	68.09	1.33	4.40

## Supplementary Materials

The following supporting information can be downloaded at: https://www.mdpi.com/article/10.3390/ma15217431/s1, Figure S1. TEM images for NaY (a), DSiY1 (b), DAlYO5 (c), and DAlYH (d); Figure S2. Pictures of the as-prepared powders under daylight and with UV irradiation; Figure S3. PL spectra for NaY-Ag, DSiY1-Ag, DSiY2-Ag, DAlYO1-Ag, DAlYO3-Ag, DAlYO5-Ag, and DAlYH-Ag; Table S1. XPS data of elemental composition of Ag+-exchanged zeolite (atomic %); Table S2: Binding energies of Ag 3d_3/2_ and Ag 3d_5/2_ in Ag^+^-exchanged zeolite.
