# Supplementary material for: Tailoring the Luminescence Properties of Silver Clusters Confined in Faujasite Zeolite through Framework Modification
Source: Materials (Basel). 2022 Oct 23;15(21):7431. doi: 10.3390/ma15217431 (PMC9657907; doi:10.3390/ma15217431)
Supplement: Supplementary file 1 [file materials-15-07431-s001.zip › materials-1954743-supplementary.pdf]

Supporting Information

Tailoring the luminescence properties of silver clusters confined in faujasite zeolite through framework modification

Xinling Xv, Song Ye,* Ling Pan, Peixuan Lin, Huazhen Liao, Deping Wang

School of Materials Science and Engineering, Tongji University, Shanghai 201804 China

Corresponding author: yesong@tongji.edu.cn

Table S1. XPS Data of Elemental Composition of Ag^{+}-exchanged Zeolite (atomic %).

Sample	XPS atomic (%)					Ag/Na Ratio
	Si	Al	O	Na	Ag	
NaY-Ag	20.86	7.84	61.02	4.72	5.56	1.18
DSiY1-Ag	19.36	9.09	59.55	5.77	6.23	1.08
DSiY2-Ag	17.06	11.39	60.13	6.90	4.52	0.66
DAlYO1-Ag	23.32	6.85	64.52	3.11	2.19	0.70
DAlYO3-Ag	24.09	6.43	65.07	2.40	2.01	0.84
DAlYO5-Ag	25.41	5.34	65.88	1.62	1.75	1.08
DAlYH-Ag	26.96	4.81	66.49	0.45	1.30	2.89

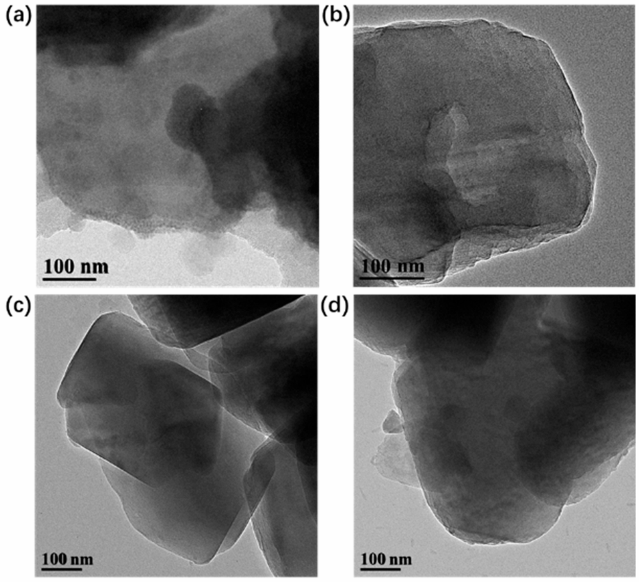

Figure. S1. TEM images for NaY (a), DSiY1 (b), DAlYO5 (c) and DAlYH (d), respectively.

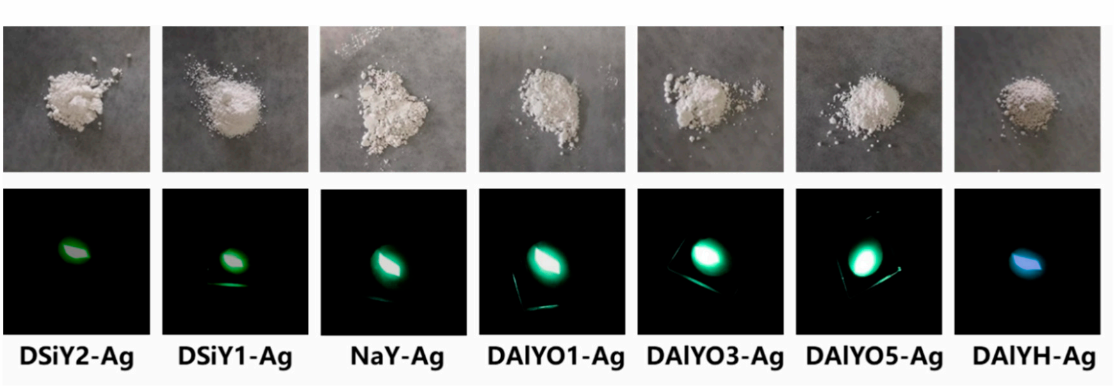

Figure. S2. Pictures of the as-prepared powders under daylight and with UV irradiation, respectively.

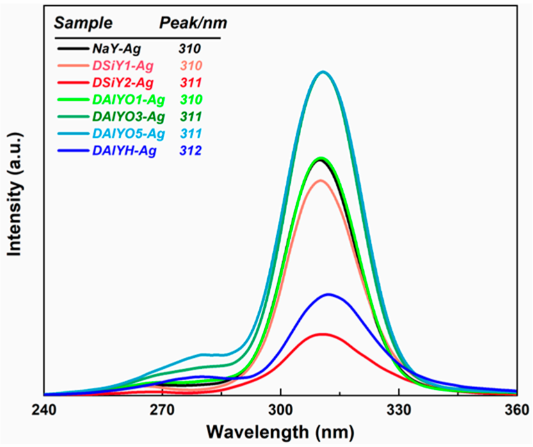

Figure. S3. PL spectra for NaY-Ag, DSiY1-Ag, DSiY2-Ag, DAlYO1-Ag, DAlYO3-Ag, DAlYO5-Ag and DAlYH-Ag, respectively.

Table S2. Binding energies of Ag $3d_{3/2}$ and Ag $3d_{5/2}$ in Ag^+-exchanged zeolites.

Samples	Ag $3d_{3/2}$ (eV)	Ag $3d_{5/2}$ (eV)
NaY-Ag	374.6	368.6
DSiY1-Ag	374.4	368.4
DSiY2-Ag	374.2	368.2
DAlYO1-Ag	374.8	368.8
DAlYO3-Ag	374.8	368.8
DAlYO5-Ag	374.8	368.8
DAlYH-Ag	374.9	368.9
